# Reviewer acknowledgement 2013

**DOI:** 10.1186/2052-1847-6-3

**Published:** 2014-01-30

**Authors:** Fernando Marques

**Affiliations:** 1 BioMed Central, 236 Gray’s Inn, London, WC1X 8HB, UK

## Abstract

**Contributing reviewers:**

The Editors of *BMC Sports Science, Medicine and Rehabilitation* would like to thank all our reviewers who have contributed to the journal in Volume 13 (2013).

## 

Chris Abbiss

Australia

Hiroshi Akima

Japan

Sivan Almosnino

Canada

Ryuzo Arai

Japan

Tiago Barbosa

Singapore

Tahsin Beyzadeoglu

Turkey

Karen Birch

UK

Quamar Bismil

UK

Shaun Boe

Canada

Richard Bohannon

US

Aurelien Bringard

Switzerland

Martin Burtscher

Austria

Michael Carmont

UK

WL Chan

Hong Kong

Birinder Cheema

Australia

Mario Costa

Portugal

Dana Crawford

US

Antonio Crisafulli

Italy

Eling D. de Bruin

Switzerland

Eamonn Delahunt

Ireland

Dennis Dolny

US

Pascal Edouard

France

Gertjan Ettema

Norway

Aharon Finestone

Israel

Steven Gaskill

US

Hannes Gatterer

Austria

Anastasios Georgoulis

Greece

Masafumi Gotoh

Japan

Lan-Yuen Guo

Taiwan

Masahiro Hasegawa

Japan

Toshiaki Hirose

Japan

Shuji Horibe

Japan

Yuichi Hoshino

Japan

Gerwyn Hughes

UK

Brendan Humphries

Australia

Fernando Idoate

Spain

Eichi Itadera

Japan

Prawit Janwantanakul

Thailand

Birgit Juul-Kristensen

Denmark

Enda King

Ireland

Kensuke Kume

Japan

Kwan-Hwa Lin

Taiwan

Francois Luthi

Switzerland

Nicola Maffulli

UK

Amelia Marques

Brazil

Tomoyuki Matsumoto

Japan

Alan McCall

France

Michael McElveen

US

William Meehan

US

Onno G. Meijer

Netherlands

Thomas Metaxas

Greece

Yukihide Minoda

Japan

Gavin Moir

US

Toshiyasu Nakamura

Japan

David C. Nieman

US

Anna Öhman

Sweden

Gøran Paulsen

Norway

Maria Francesca Piacentini

Italy

Stelios Psycharakis

UK

Greg Robertson

UK

James Rupert

Canada

Daniel Sánchez-Zuriaga

Spain

Kimi Sato

US

Christophe Schnitzler

France

Aaron Sciascia

US

Roberto Seijas

Spain

Jasper Shealy

US

Arthur Siegel

US

Goran Sporis

Croatia

Siobhan Strike

UK

Antoni Sureda

Spain

Hideki Takagi

Japan

Harukazu Tohyama

Japan

Erik Wikstrom

US

Del P. Wong

Hong Kong

Jari Ylinen

Finland

Natasa Zenic

Croatia

## Pre-publication history

The pre-publication history for this paper can be accessed here:

http://www.biomedcentral.com/2052-1847/6/3/prepub

